# Citrullination of Proteins as a Specific Response Mechanism in Plants

**DOI:** 10.3389/fpls.2021.638392

**Published:** 2021-04-08

**Authors:** Claudius Marondedze, Giuliano Elia, Ludivine Thomas, Aloysius Wong, Chris Gehring

**Affiliations:** ^1^Division of Biological and Chemical Science and Engineering, King Abdullah University of Science and Technology, Thuwal, Saudi Arabia; ^2^Rijk Zwaan, De Lier, Netherlands; ^3^Department of Biochemistry, Faculty of Medicine, Midlands State University, Gweru, Zimbabwe; ^4^Conway Institute of Biomolecular and Biomedical Research, University College Dublin, Dublin, Ireland; ^5^Department of Biology, College of Science and Technology, Wenzhou-Kean University, Wenzhou, China; ^6^Zhejiang Bioinformatics International Science and Technology Cooperation Center of Wenzhou-Kean University, Wenzhou, China; ^7^Department of Chemistry, Biology and Biotechnology, University of Perugia, Perugia, Italy

**Keywords:** citrullination, citrullinome, post-translational modification, arginine deiminase, cold stress

## Abstract

Arginine deimination, also referred to as citrullination of proteins by L-arginine deiminases, is a post-translational modification affecting histone modifications, epigenetic transcriptional regulation, and proteolysis in animals but has not been reported in higher plants. Here we report, firstly, that *Arabidopsis thaliana* proteome contains proteins with a specific citrullination signature and that many of the citrullinated proteins have nucleotide-binding regulatory functions. Secondly, we show that changes in the citrullinome occur in response to cold stress, and thirdly, we identify an *A. thaliana* protein with peptidyl arginine deiminase activity that was shown to be calcium-dependent for many peptide substrates. Taken together, these findings establish this post-translational modification as a hitherto neglected component of cellular reprogramming during stress responses.

## Introduction

**FIGURE 1:**
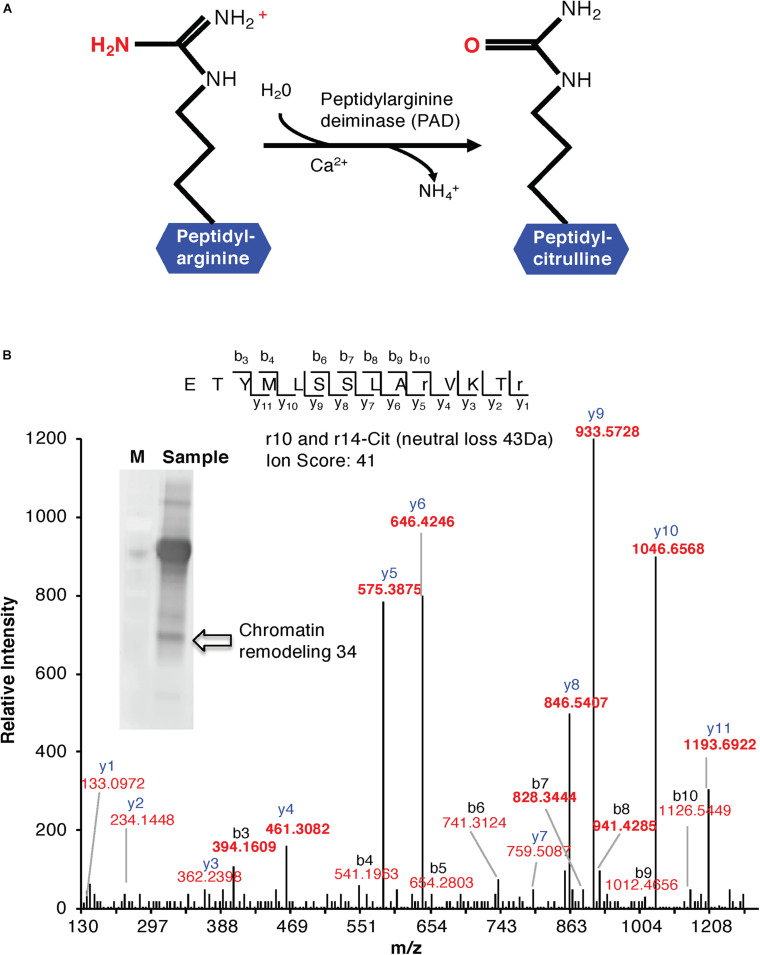
**(A)** Enzymatic formation of a citrulline. Enzymatic hydrolysis of peptidyl-arginine to peptidyl-citrulline by peptidylarginine deiminase (PAD). **(B)** MS/MS spectra of a citrullinated Chromatin Remodeling 34 (ChR34). The MS/MS spectrum of Chromatin Remodeling 34 (At2g21450) contains citrullinated residues on positions 10 and 14. Indicated on the peptide sequence are the detected fragment ions, as indicated by the *b*- and *y*-ions and their relative intensities. The ion score of the peptide is 41. The insert shows a western blot of anti-citrulline immunoprecipitated proteins. The arrow shows the protein band identified as “Chromatin Remodeling 34.”

Citrulline, an intermediate of the urea cycle, is not comprised among the 20 amino acids that constitute the building blocks of the proteins. However, it can be formed in proteins and peptides as a result of arginine deimination (Figure 1A). The presence of citrulline in protein extracts was first reported well over half a century ago in the red alga *Chondrus crispus* (Smith and Young, 1955). It was later discovered in hair follicles and established unequivocally in peptide linkages (Rogers and Simmonds, 1958; Rogers, 1962). Citrullination by agmatine deiminases was also reported in bacteria (for review, see Linsky and Fast, 2010), and it was shown that the enzyme is a member of the large family of guanidino-group modifying enzymes that catalyze a variety of reactions, including guanidinium hydrolysis and amidino group transfer. Other members of the family are amidinotransferases, dimethylarginine dimethylaminohydrolase, L-arginine deiminase, and protein arginine deiminases.

The enzymatic conversion of an arginine residue into a citrulline in a protein results in the loss of a positive charge that perturbs a number of intra- and intermolecular electrostatic interactions causing partial unfolding of the citrullinated protein (Tarcsa et al., 1996). Consequently, these may become more prone to protease attack and may become immunogenic. A disturbance of the cellular citrullination rates in proteins has been shown to play a central role in the pathogenesis of a number of autoimmune and neurological disorders such as rheumatoid arthritis (van Venrooij and Pruijn, 2000; Klareskog et al., 2013). Citrullination is also important in physiology. Examples include terminal differentiation of the epidermis, apoptosis or the modulation of brain plasticity during postnatal life, as well as the regulation of pluripotency (Christophorou et al., 2014) and cancer (Yuzhalin, 2019; for recent reviews, see Beato and Sharma, 2020; Brentville et al., 2020; Fert-Bober et al., 2020).

Importantly, citrullination has been reported to have a role in the epigenetic regulation of gene transcription (Thompson and Fast, 2006). In several species, histones H2A, H3, and H4 have been shown to be citrullinated by the peptidyl arginine deiminase type IV (PAD4) in the nucleus of granulocytes where the reaction uses both arginine and methylarginine (but not dimethylarginine) as substrates. It has been hypothesized that arginine demethylation by PAD4 might act to “reverse” the transcriptional coactivator function of the protein arginine methyltransferases (PRMTs) by hydrolyzing methylated arginines to citrulline (Arita et al., 2006; Thompson and Fast, 2006). PAD-catalyzed hydrolysis of methylated arginines would not truly reverse the modification but instead leave peptidylcitrulline as a final product.

To date, reversibility by an arginine demethylase has not been reported. However, the PAD4 enzyme, which can remove the methyl groups introduced by the PRMTs, might serve an analogous function, possibly together with as yet undiscovered transaminases, thereby restoring arginine groups in histones. The structural basis of PAD4 activation by Ca^2++^ in humans has also been reported (Arita et al., 2004).

Peptidyl arginine deiminase s have been discovered in several mammalian species and in other vertebrates, like birds and fish, but have not been observed in bacteria, lower eukaryotes, or higher plants (György et al., 2006; El-Sayed et al., 2019). A notable exception is the anaerobic human pathogen *Porphyromonas gingivalis* that harbors an enzyme termed PPAD that shows little sequence similarity to the human PAD enzymes but can convert both peptidyl arginine and free arginine to peptidylcitrulline and free citrulline, respectively. However, PPAD appears to be Ca^2+^-independent (György et al., 2006).

In plants, the presence of the non-proteinogenic amino acid citrulline was known for over a century (for review, see Joshi and Fernie, 2017). More recently, many aspects of its metabolism and localization, transport, and accumulation have been elucidated (Joshi and Fernie, 2017). However, citrullination as a post-translational modification catalyzed by PADs has not been seriously considered, and, in fact, it has been suggested that in the absence of PAD function *in planta*, the regulation of protein function through citrullination was an unlikely proposition (for review, see Gudmann et al., 2015).

Here we asked the following questions: (1) Is there evidence for citrullinated proteins in *Arabidopsis thaliana*, and if so, (2) are citrullination signatures stimulus-specific, and (3) what are plausible *A. thaliana* deiminase candidates that can citrullinate proteins?

## Results and Discussion

### Identification of Citrullinated Peptides and Proteins in *Arabidopsis thaliana*

Cell suspension cultures derived from the roots of *A. thaliana* (ecotype Columbia-0) were used in this study to examine the presence of citrullinated arginines in higher plants. Cells (control) were subjected to nuclear fraction enrichment, and citrullinated proteins were enriched by immunoprecipitation followed by western blotting (Supplementary Figure 1). Given the novel promising results from western blot analysis and the relatively uncharacterized nature of the anti-citrullinated protein antibody in plant systems, we validated the results by tandem mass spectrometry (LC-MS/MS) following in-blot digestion or direct in-solution digestion of the immunoreactant proteins. Realizing the potential weaknesses that may arise with westerns specifically to distinguish unequivocally between peptidylcitrulline (from peptidylarginine) and homocitrulline (from carbamylation of lysine; Verheul et al., 2017), we took advantage of the more discriminating tandem mass spectrometry and manually checked the spectra to verify that citrullinated amino acid in a peptide corresponded to the respective arginine in the unmodified peptide. The LC-MS/MS analysis shows that citrulline was detected on proteins from the immunoreactant samples including the detection of the citrullinated peptide of chromatin remodeling 34 (CHR34; At2g21450; Figure 1B). Here we noted that citrullination was rather targeted and to specific arginine residues, for example, only 2 of the 52 arginine residues were citrullinated in CHR34. Besides, a total of 14 citrullinated peptides, corresponding to 14 individual proteins, were identified from the control samples. Ten of these did not show any changes in response to stimuli (described below) and are considered a baseline citrullination signature of the nuclear enriched proteome in our sample (Table 1). One of the citrullinated proteins, the target of Rapamycin (TOR) related protein (At1g50030), belongs to the family of phosphatidylinositol 3-kinase. They are targets of the antiproliferative drug rapamycin and are annotated as having a role in many processes, including metabolic reprogramming (Masui et al., 2014), transcription and rRNA processing, and protein self-association. In addition, it may also contribute to ending seed dormancy. TOR is also a DNA-binding protein (Table 1). Other notable DNA-binding proteins identified include far-red impaired responsive protein (FAR1; At4g12850) and methyltransferase MT-A70 (At4g09980). The FAR1 is a transcription factor co-opted from ancient mutator-like transposase, which is located in the nucleus and is a component of phytochrome A mediated far-red light signaling (Wang and Wang, 2015). The extensive similarity of FAR1 with the mutator-like transposases points to a multitude of roles for FAR1 in developmental and physiological processes including stress responses, programmed cell death, reactive oxygen species (ROS) homeostasis, and abscisic acid (ABA) signaling and branching. These diverse roles of FAR1 may implicate citrullination in modulating DNA-binding, light signaling, and stress responses. However, with methyltransferase MT-A70, citrullination may be linked to seed dormancy and embryogenesis. Methyltransferase MT-A70 is an S-adenosylmethionine-dependent methyltransferase with a role in seed dormancy release, which, in turn, is controlled by the balance between ABA, GA, and ethylene (Linkies et al., 2009). The methyltransferase MT-A70 gene is also strongly upregulated in suspension culture cells exposed to cold (4°C) for 24 h and then to cordycepin (3′-deoxyadenosine, an inhibitor of transcription) 1 h prior to harvesting cells. Furthermore, a ubiquitin associated protein, RING/FYVE/PHD zinc finger protein (At3g02890), is also involved in DNA methylation, RNA interference, and the biological process of methylation-dependent chromatin silencing. However, the role of citrullination on all these biological processes is yet to be established.

**TABLE 1:** Citrullinated peptides and low-temperature stress induced citrullination events in *Arabidopsis thaliana* proteins.

Accession number	Description	Mascot score	Mass	Peptide (Pep.)	Pep. Score (≥30)	Expect	FC C vs 1 h	FC C vs 24 h
**Citrullinated peptides in the control:**								
AT1G50030^#^	Target of rapamycin (TOR)	59	281237	R.ERAVEALR.A	59	0.0003	ns	ns
AT1G26110^#^	Decapping 5 (DCP5)	47	64331	R.GYGGyGGRGGGGGGyGyGGRGQGR.G	47	0.007	ns	ns
AT5G17860^#^	Calcium exchanger 7 (CAX7)	40	62679	K.RMSDQILR.S	39	0.033	ns	ns
AT4G12850^#^	Far-red impaired responsive (FAR1)	37	21522	K.VRDIVENVKK.L	36	0.035	ns	ns
AT4G31890^#^	Armadillo (ARM) repeat protein	35	56650	R.VtLAMLGAIPPLVSmIDDsR.I	35	0.0097	ns	ns
AT4G27810^#^	Hypothetical DUF688 protein	34	22156	R.RSLSVIR.R	34	0.031	ns	ns
AT3G02890^#^	RING/FYVE/PHD zinc finger	33	110280	R.RVGNRPMGRR.G	33	0.022	ns	ns
AT4G00830^#^	RNA-binding family protein	33	55273	R.NNGSSGGSGRDNSHEHDGNRGGR.R	33	0.027	ns	ns
AT4G09980^#^	Methyltransferase MT-A70	32	86022	R.ERTHGSSSDSSK.R	32	0.024	ns	ns
AT4G10000^#^	Thioredoxin family protein	30	37375	R.ISGNGNWVRER.R	30	0.018	ns	ns
**Peptides differentially citrullinated in response to cold treatment:**								
AT2G21450*	Chromatin remodeling 34 (CHR34)	68	94037	K.ETYmLSSLARVKTR.R	41	0.0018	1.90	1.84
AT5G27920^#,^	*F*-box family protein	41	72636	R.ARGLETLAR.M	47	0.0029	1.86	1.53
AT4G19180*	GDA1/CD39 nucleoside phosphatase	37	82544	R.FQRWSPMSTGVK.T	37	0.0077	1.9	1.8
AT1G29040^#^	Hypothetical exostosin family prot.	33	20111	K.FLSSRSK.Q	33	0.013	ns	0.67
***De novo* cold-induced citrullinated peptides:**								
AT1G24290^#^	AAA-type ATPase family protein	37	57702	K.SMRGGDANAAIYWLAR.M	35	0.013	at 1 h	at 24 h
ATCG00520	YCF4 (unfolded protein binding)	35	21564	K.DIQsIRIEVK.E	35	0.014	at 1 h	nd
AT1G16950	Hypothetical protein	34	10044	.MARPRIsIsmICLLILIVGFVLQssQAR.K	34	0.0071	nd	at 24 h
AT2G23790	Unknown DUF607 protein	32	38438	K.LLRAAQIEIVK.S	32	0.02	at 1 h	nd
AT2G47650	UDP-xylose synth. 4	32	50085	R.VVVtGGAGFVGSHLVDRLmAR.G	32	0.0087	nd	at 24 h

Citrulline candidate proteins indicating their accession numbers, description, Mascot scores, citrullinated peptide(s) with the citrullinated residue highlighted in red, peptide ion scores, expect, and fold changes (FC) following cold treatment. *Identified by gel-based and gel-free techniques. ^#^Identified by gel-based technique. ns, not significant at *p* ≤ 0.05 and FC = 0.68 < *x* > 1.5; nd, not detected. Letters in red denote citrullinated arginine residues; lower case “y,” “t,” and “s” indicate phosphorylated residues and “m” are oxidized methionines.

Protein decapping 5 (DCP5; At1g26110), one of the three RNA-binding proteins identified with a citrullinated peptide, is a positive regulator of cytoplasmic mRNA and a negative regulator of translation (Xu and Chua, 2009). In addition to having protein binding and protein homodimerization activities, it is transcriptionally upregulated by the herbicide primisulfuron-methyl. DCP5 has been identified as a candidate protein interacting with the *A. thaliana* Cold Shock Domain Protein 3 (AtCSP3) that shares an RNA chaperone function with Escherichia *coli* cold shock proteins and regulates freezing tolerance during cold acclimation (Kim et al., 2013). Like DCP5, LHP1-INTERACTING FACTOR 2 (LIF2, At4g00830) is an RNA-binding protein that encodes a heterogeneous nuclear ribonucleoprotein that is involved in the plant innate immune response and may function as a suppressor of cell-autonomous immunity (Molitor et al., 2016). LIF2 is involved in cell identity and cell fate decision and has been postulated to modulate the activity of LHP1, a polycomb repressive complex 1 subunit, at specific loci, during specific developmental windows or in response to environmental cues that control cell fate determination (Latrasse et al., 2011). Furthermore, a transcriptional LIF2 response^1^ to some pathogens and elicitors was noted, and this may implicate citrullination in the defense against biotic challenges. Taken together, key functional roles of LIF2 correlate well with the role of citrullination described in the animal systems (Christophorou et al., 2014) and may suggest an expanding role of citrullination in plant development and stress responses.

Three proteins were associated with protein binding activities, and these include armadillo (ARM)-repeat protein (At4g31890), YCF4 (ATCG00520), and thioredoxin family protein (At4g10000). Armadillo protein is part of a large family with diverse functions in many eukaryotes. It has been proposed that the structure of ARM-repeats enables functional versatility (Tewari et al., 2010). The tandem ARM-repeat can fold itself to form a superhelix that can enable protein–protein interactions and, in turn, activate or modulate signaling networks. It is therefore conceivable that citrullination could affect cellular signaling events via armadillo citrullination. On the other hand, thioredoxins are small disulfide-containing proteins that have electron carrier activity and participate in redox homeostasis (Meyer et al., 2008; Bertoni, 2011). Plant thioredoxins can act as antioxidants by activating ROS scavenging enzymes and regulate the activity of key enzymes in processes such as photosynthetic carbon fixation and anther dehiscence (Meyer et al., 2008). In addition, thioredoxins have been shown to activate their target enzymes by reducing Cys disulfides (Montrichard et al., 2009). Considering that most of the proteins detected in this study are linked to stress events, it is conceivable that the role of thioredoxins in ROS activity may be modulated by citrullination. In addition, calcium exchanger 7 (At5g17860) is also linked to stress responses. Calcium exchanger 7 is a Ca^2+^:Na^+^ antiporter that is highly expressed during senescence and in response to fungal infection (Shigaki and Hirschi, 2006).

### Stimulus-Specific Changes in the Citrullination Signature

In order to detect possible stimulus-specific changes in citrullination signatures, we decided to test the effects of exposure of cells to low temperature and this for two reasons: firstly, because the arabidopsis agmatine iminohydrolase is itself transcriptionally regulated by cold [see https://genevestigator.com/gv/ (Zimmermann et al., 2004)], and secondly, temperature changes can be applied easily and reproducibly to suspension culture cells. In response to cold, four proteins showed differential expression and abundance in their citrullination signature over time (0, 1 h, and 24 h; Table 1). These proteins include the chromatin remodeling 34 (CHR34; At2g21450; Figure 1B) that shows an increase in citrullination after cold stress at both 1 and 24 h. CHR34 is strongly transcriptionally upregulated by several herbicides including primisulfuron-methyl and cloransulam-methyl and sulfometuron-methyl [see https://genevestigator.com/gv/ (Zimmermann et al., 2004)]. In addition, citrullination of three proteins, F-box family protein (At5g27920), GDA1/CD39 nucleoside phosphatase (At4g19180), and hypothetical exostosin family protein (At2g29040), increased in response to cold treatment, and these proteins are expressed in the male gametophyte, pollen tube, and flower. The F-box protein is annotated as having ubiquitin-protein transferase activity. F-box proteins contain a conserved motif (40–50 amino acids) at their *N*-terminal that functions as a site of protein–protein interactions. F-box protein components of the Skp I-Cullin-F-box complex are critical for regulating cell signaling as well as controlling the degradation of cellular proteins (Jain et al., 2007). The F-box protein-encoding genes are influenced by light in addition to other abiotic stress conditions as shown by the differential expression of at least 43 F-box protein-encoding genes in rice (*Oryza sativa*) seedlings (Jain et al., 2007). The GDA1/CD39 nucleoside phosphatase (At4g19180) is annotated as expressed in cultured plant cell and the flower, and in particular the gynoecium, pollen, and sepal.

Furthermore, five proteins undergo *de novo* citrullination in response to cold stress. For the AAA-type ATPase (At1g24290), citrullination is observed at both 1 and 24 h after the onset of cold stress conditions (Table 1). The protein is annotated as playing a role in DNA replication and is most highly expressed in the carpel and the seed, and during senescence. It is noteworthy that the gene is transcriptionally upregulated in response to cold, while it is repressed in response to ABA [see https://genevestigator.com/gv/ (Zimmermann et al., 2004)]. The YCF4 chloroplast protein (ATCG00250) and the hypothetical protein (At1g16950) are citrullinated only after 1 h of cold treatment (Table 1). YCF4 is annotated as having a role in transcription, DNA template, and elongation, as well as in “unfolded protein binding” and the assembly of photosystem II. It may also act in translational elongation and triplet codon-amino acid adaptor activity (Heyndrickx and Vandepoele, 2012). The hypothetical protein (At1g16950) is citrullinated only 24 h after cold treatment and is also expressed during the flowering stage and in particular in the stamen. The most pronounced transcriptional increase (>8-fold) occurs in double mutants of the transcription factors Auxin Response Factor 6 and 8 (ARF 6 and 8) that activate jasmonate biosynthesis. It is also upregulated in severely gibberellic acid (GA) deficient mutants (*ga1-3*) that are dwarfed and male sterile [see https://genevestigator.com/gv/ (Zimmermann et al., 2004)]. The unknown DUF607 (At2g23790) is citrullinated after only 1 h of cold treatment (Table 1) and is again highly expressed in flowers and seedlings. This unknown protein is also referred to as the mitochondrial inner membrane calcium uniporter, and it mediates calcium uptake into mitochondria (Stael et al., 2012). Finally, the UDP-xylose synthase 4 (At2g47650) is citrullinated only after prolonged exposure to cold (24 h), and cold stress causes marked (>2-fold) transcript accumulation. Overall, we noted that the majority of the proteins that are differentially citrullinated in response to low-temperature stress are linked to general stress responses and importantly reproductive stages of plant development including key organs such as flower and pollen. This is indicative of the vital role that citrullination may have in stress adaptation during this critical stage in the plant development.

When we used Weblogo, a software package that extracts specific signatures in a set of proteins, it appears that citrullinated arginine residues have some preferential neighbors (see Supplementary Figure 2). On the *N*-terminal, two amino acids removed from the citrullinated arginine, we see a preference for serine or arginine; on the *C*-terminus, four amino acids removed from the citrullinated arginine, we noted a preference for an arginine and five amino acids removed a leucine. If we translate this into a search pattern [RS]x**R**x{4}RL, where **R** is the target of citrullination, and query the *A. thaliana* proteome, we find that it occurs in >900 proteins. In addition, we observe an enrichment of glycine within the amino acids neighboring the citrullinated **R**, conceivably for affording structural stabilization of the substrate for citrullination. Finally, given that noncitrullinated arginines are found in the vicinity of the citrullinated arginine is a further indication for the topological specificity of citrullination events.

### In Search of Candidate Deiminases in *Arabidopsis thaliana*

We first checked if the arabidopsis proteome contains any orthologues of annotated deiminases and noted that an arabidopsis agmatine iminohydrolase (At5g08170) has also been annotated as porphyromonas-type peptidyl-arginine deiminase family protein containing agmatine deiminase (InterPro:IPR017754) and peptidyl-arginine deiminase of the Porphyromonas-type (IPR007466) sites. Crystal structures representing the Michaelis complex and the thiouronium reaction intermediate of *Pseudomonas aeruginosa* arginine deiminase (Galkin et al., 2005) as well as an arabidopsis agmatine iminohydrolase (Levin et al., 2007) have been elucidated. The predicted highly conserved catalytic residues of the peptidylarginine deiminase (Q9RQJ2) from *P. gingivalis* include Asp130, His236, Asp238, and Cys351 and an Asp187 that is absent in two family members of iminohydrolase families (Shirai et al., 2001; Marchler-Bauer et al., 2015). The arabidopsis iminohydrolase, in turn, does contain these conserved residues, the Asp169, His224, Asp226, and Cys366 residues diagnostic for peptidyl-arginine deiminases and notably the aspartates that engage in ionic interactions with the guanidinium group in the cysteine.

**FIGURE 2:**
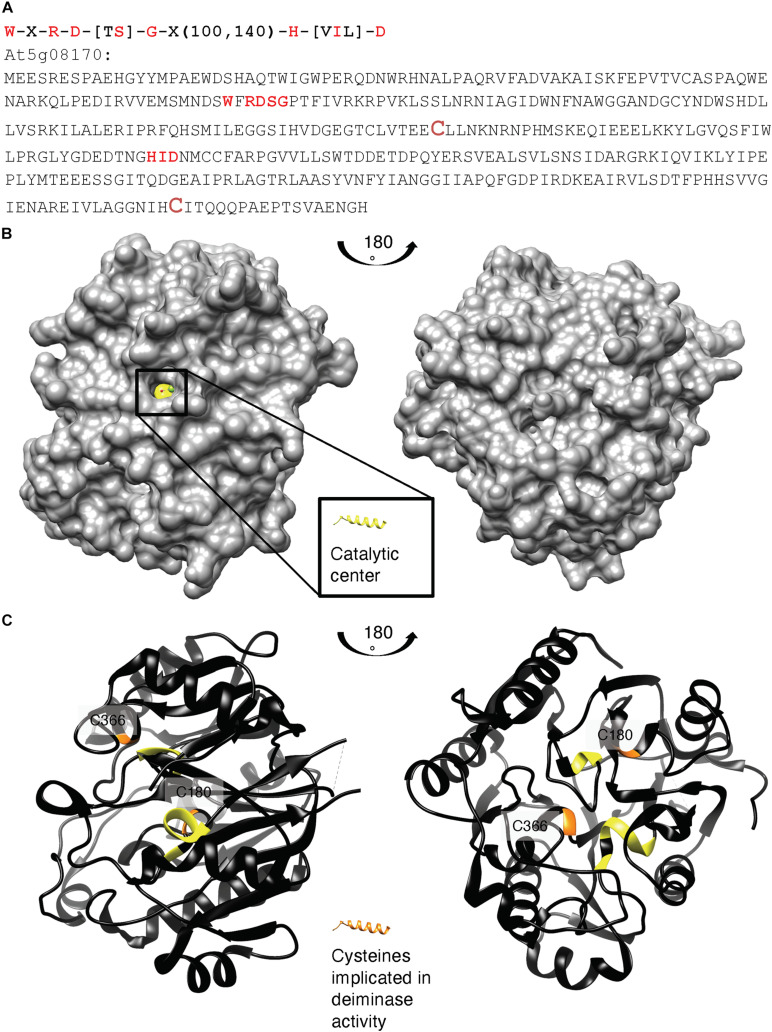
**(A)** Amino acid sequence, **(B)** surface, and **(C)** ribbon structure of At5g08170 (PDB ID: 3H7K). Amino acids appearing in the motif: W-X-R-D-[TS]-G-X(100,140)-H-[VIL]-D are highlighted in red in **(A)** the full-length At5g08170 amino acid sequence, and colored yellow in **(B)** the surface and **(C)** ribbon At5g08170 structures. The cysteines (C180 and C366) implicated in the catalytic activity of agmatine deiminase are highlighted in brownish red in **(A)** and orange in **(C)**, the ribbon structures. Amino acids R93–G96 from the motif occupy a clear cavity that can spatially accommodate an R residue **(B)** and thus is assigned the catalytic center for arginine deiminase (highlighted in yellow). All analyses and images were created using UCSF Chimera (ver. 1.10.1). Chimera was developed by the Resource for Biocomputing, Visualization, and Informatics at the University of California, San Francisco (supported by NIGMS P41-GM103311).

We also aligned key amino acid residues in the catalytic center (Wong et al., 2015, 2018) of annotated bacterial agmatine deiminases and created a search motif: W-X-R-D-[TS]-G-X(100,140)-H-[VIL]-D (Figure 2A). This motif identified At5g08170 as a sole protein in the *A. thaliana* proteome. Highly homologous sequences do also occur in other eudicots (e.g., the *Camelia sativa*: 98% cover, *e*-value: 0.0) and naturally the ancestral bacterial agmatine deiminases (e.g., *Pseudomonas stutzeri*: 97% cover, *e*-value: 5e-144) but not in vertebrates. An alignment is shown in Supplementary Figure 3.

Based on the crystal structure of At5g08170 (PDB ID: 3H7K), the key amino acids appearing in the motif were located. Notably, these conserved amino acids constitute a clear cavity (highlighted in yellow) that is solvent exposed (Figure 2B) and that is also spatially able to accommodate an arginine residue. This cavity is thus assigned the catalytic center for arginine deiminase (Figures 2B,C).

Next, we evaluated if arginines in two selected citrullinated proteins (At2g21450 and At4g00830) identified from cell cultures of arabidopsis roots can dock at the assigned catalytic center of At5g08170. In order to perform docking simulations, models of both citrullinated proteins were first generated by homolog modeling against the chain K of an ATPase domain of a chromatin remodeling factor (PDB ID: 6PWF) and the chain D of decaheme c-type cytochrome (PDB ID: 6R2K), respectively. Based on the 3D models, the citrullinated arginines (colored according to surface charges) were determined to be solvent-exposed and protruding into space that is visibly large enough for the arginines to have unimpeded interactions with the catalytic center of At5g08170 (Supplementary Figures 4A,B). The individual citrullinated residues—R427 and R431 of At2g21450, and R477 and R487 of At4g00830—were, respectively, docked at the catalytic center cavity of At5g08170, keeping all bonds in the R ligand nonrotatable so that their poses from the generated 3D models are preserved. All citrullinated arginines docked at the catalytic cavity in a binding pose deemed suitable for catalysis, i.e., with the amine-rich region pointing into the cavity, as shown in the surface models (right panels; Supplementary Figures 4B,C).

### *In vitro* Citrullination Activity of At5g08170

*In vitro* citrullination involving recombinant agmatine deiminase was performed using various substrates including fibrinogen (Sigma-Aldrich, St. Louis, MO, United States) and peptides generated from LIF2 and chromatin remodeling 34 (Table 1). Fibrinogen has been shown to be a target of citrullination by agmatine deiminase from *P. gingivalis* (Lundberg et al., 2010) and was also citrullinated by the recombinant arabidopsis agmatine deiminase (Supplementary Table 1). This suggests broad substrate specificity of the enzyme. As an aside, fibrinogen citrullination has recently been demonstrated to affect the rate of fibrin polymerization (Damiana et al., 2020), thereby impairing the fibrin clot structure, which, in turn, is likely to have direct implications for the inflammatory response.

We then tested whether the recombinant enzyme could citrullinate peptides from proteins identified by mass spectrometry as potential citrullination candidates. Using LIF2 and chromatin remodeling 34, we could show that agmatine deiminase can citrullinate the LIF2 peptide (Supplementary Table 2). However, citrullination of the chromatin remodeling 34 peptide had a low confidence score, and we postulated that this could be attributed to the peptide failing to form the needed structural conformation. Since it has been shown that the orthologs of agmatine deiminase can undergo auto-citrullination (Andrade et al., 2010), we tested if this is the case for the arabidopsis agmatine deiminase. When we tested auto-citrullination in the presence or absence of Ca^2+^, we observed that agmatine deiminase auto-citrullination does occur and is many cases more pronounced in the presence of Ca^2+^ (Supplementary Table 2). There were a total of 14 possible Ca^2+^ binding sites in At5g08170 predicted by the MIB: Metal Ion-Binding site prediction and docking server available at http://bioinfo.cmu.edu.tw/MIB, with binding residues P97, G117, and D119 forming the binding site that is closest to the catalytic center (R93–G96; Supplementary Figure 5). Since this binding site occupies the region at the opening of the cavity, it could modulate the catalytic activity, thus accounting for the more pronounced auto-citrullination activity.

### Concluding Remarks

We conclude from this that citrullination events do occur in higher plants, particularly in arabidopsis, and that the arabidopsis proteome does contain proteins that incur citrullination. In addition, low-temperature stress causes stimulus-specific protein citrullination of a set of proteins, many of which have annotated roles in stress responses. Furthermore, our data also suggest that citrullination may be part of the general regulation of pluripotency. Finally, we also predict that specific citrullination could occur in response to biotic and abiotic stimuli and that stress-specific citrullination signatures and their downstream effects influence plant growth, development, and stress responses.

## Materials and Methods

### Arabidopsis Cell Suspension Culture

Cells derived from the roots of *A. thaliana* (ecotype Columbia-0) were grown in 100 mL of Gamborg’s B-5 (Gamborg and Eveleigh, 1968) basal salt mixture (Sigma-Aldrich, St Louis, MO, United States) with 2,4-dichlorophenoxyacetic acid (0.5 μg mL^–1^) and kinetin (0.05 μg mL^–1^) in 250 mL sterile flasks in a growth chamber (Innova^®^ 43, New Brunswick Scientific Co., NJ, United States) at 120 rpm, with photosynthetic light set for 12 h light/12 h dark cycles at 23°C, and subcultured every 7 days (Turek et al., 2014; Marondedze et al., 2015; Piazza et al., 2015). Cells were cold (4°C) treated and collected at 0, 1, and 24 h post-treatment. Media was drained off using a Stericup^§^ filter unit (Millipore, Billerica, MA, United States), and cells were immediately frozen in liquid nitrogen and stored at -140°C until further use.

### Nuclear Protein Extraction

Nucleus enrichment was performed using a nucleus enrichment kit for tissue (Pierce, Rockford, IL, United States). Briefly, protease inhibitors were added to the appropriate volume of nucleus enrichment reagents A and B. A total of 500 mg of frozen callus cells was transferred to a 50 mL Falcon tube, and 800 μL of reagent A and 800 μL of reagent B were added. The tissue was homogenized for 4 s twice on ice using a PowerGen 125 grinder (Fisher Scientific, Rockford, IL, United States). The homogenate was transferred to a fresh 2 mL microcentrifuge tube, mixed by inverting the tube five times and centrifuged at 500 × *g* for 10 min at 4°C to collect the supernatant. A discontinuous density gradient was prepared by carefully overlaying three OptiPrep^TM^ gradients [the first layer consisted of 1.5 mL of 27.5% (v/v), the second consisted of 1.5 mL of 23% (v/v), and the third consisted of 1 mL of 20% (v/v)]. Gradients were prepared from the OptiPrep media and a gradient dilution buffer. The tissue extract was mixed with 60% (v/v) OptiPrep^TM^ cell separation media and diluted to a final density gradient of 7.5% (v/v) with a gradient dilution buffer and overlaid on top of the density gradients. Ultracentrifugation was performed at 40,000 × *g* for 90 min at 4°C. The top 2 mL, containing the nuclei, was pipetted out and precipitated with ice-cold acetone overnight at -20°C. Proteins were pelleted at 3,900 × *g* for 15 min at 4°C, washed three times in 80% (v/v) ice-cold acetone, and solubilized in a urea lysis buffer (7 M urea, 2 M thiourea) as described in Marondedze et al. (2015). Protein concentration was estimated by Bradford (1976). Approximately 200 μg of total nucleus protein extract was reduced with 5 mM dithiothreitol, alkylated, and used for protein digestion with sequencing-grade modified trypsin (Promega, Madison, WI, United States). The resulting peptides were purified using a Sep-Pak Vac tC18 100 mg cartridge (Waters, Milford, MA, United States), as described previously (Groen et al., 2013), completely dried in a Speed Vac concentrator (Thermo Scientific, Bremen, Germany) and analyzed by tandem mass spectrometry (LC-MS/MS).

### Immunoprecipitation, Western Blot, and Quantitative Analysis of Citrullinated Proteins

Immunoprecipitation was performed using two protocols. In the first experiment, immunoprecipitation was done using the immunoprecipitation kit Dynabeads^®^ Protein A (Life technologies) and according to the manufacturer’s instruction. Here, Dynabeads^®^ were resuspended by vortexing for >30 s and 50 μL of Dynabeads^®^ was pipetted to a 1.5 mL microcentrifuge tube. The supernatant was removed using a magnetic Eppendorf tube rack prior to adding 2 μg of anti-citrulline antibody (ACAb, catalogue # ab240908; Abcam, Cambridge, United Kingdom) diluted in 200 μL phosphate buffered saline (PBS) containing Tween-20. The beads-Ab mix was incubated with rotation for 10 min at room temperature. The supernatant was removed by placing the tube on the magnetic rack, and the beads-Ab complex was resuspended in a 200 μL PBS/Tween-20 buffer, resuspended by gentle pipetting; the supernatant was removed as stated previously. The protein sample (200 μg) was then added to the beads-Ab and gently pipetted to the mix, incubated with rotation for 20 min at room temperature and supernatant decanted. The beads-Ab-protein mix was washed three times using 200 μL of a washing buffer for each wash and resuspended in a 100 μL washing buffer by gentle pipetting and transferred to a fresh tube. The supernatant was removed by means of a magnetic rack. A 20 μL elution buffer was gently pipetted to resuspend the Dynabeads^®^-Ab-protein complex and incubated with rotation for 2 min at room temperature to dissociate the complex.

In the second experiment, 200 μg of protein sample and 2 μL of anti-citrulline antibody in TBS were mixed and incubated overnight at 4°C with gentle rocking. Dynabeads^®^ Protein A (Novex^®^, Life technologies) were then added and incubated for 3 h at 4°C with gentle rocking. The protein–antibody–beads complex was washed five times with a 1 × lysis buffer diluted in TBS, resuspended in 20 μL of an elution buffer, mixed gently, and incubated with end-over-end mixing for 2 min at room temperature to dissociate the complex. The supernatant was collected using a magnetic rack.

For both experiments, the protein solution collected was split into two 10 μL aliquots; one was used for in-solution digestion and tandem mass spectrometry, and the remaining 10 μL was mixed with an equal volume of a 2 × reducing buffer for the preparation prior to one-dimensional gel electrophoresis and western blot analysis. Proteins were transferred from polyacrylamide gel to polyvinylidine difluoride (PVDF) membranes as described by Towbin et al. (1979) using a *Trans*-blot electrophoretic transfer cell (Bio-Rad). The PVDF membrane with transferred proteins was blocked overnight at 4°C in a blocking solution [5% (w/v) bovine serum albumin in TBS], washed three times with a TBS buffer containing 0.05% (v/v) Tween 20 (TBST) for 5 min, and incubated with anti-citrulline (primary antibody) diluted in TBST for 1 h at 37°C. Membranes were washed three times in PBST for 5 min, incubated with the secondary antibody, Alexa Fluor^®^ 488 goat anti-rabbit IgG (Molecular Probes, Eugene, OR, United States), diluted in TBS for 2 h at room temperature, thoroughly washed in TBST, and imaged with a Typhoon^TM^ 9410 scanner (GE Healthcare). Quantitative analyses and the relative-fold changes for protein bands were computed using the ImageQuant-TL 7.0 software (GE Healthcare). Membranes were stained using the MemCode reversible stain according to the manufacturer’s instructions (Pierce). All visibly stained bands were cut, digested with trypsin, and analyzed by LC-MS/MS.

### Tandem Mass Spectrometry and Database Search

Peptides were analyzed by mass spectrometry as described previously (Marondedze et al., 2013). Briefly, peptides were resuspended in 5% (v/v) acetonitrile and 0.1% (v/v) formic acid prior to identification by an LTQ Orbitrap Velos mass spectrometer (Thermo Electron, Bremen, Germany) coupled with an Ultimate 3000 UPLC (Dionex-Thermo-Scientific) for nano-LC-MS/MS analyses. A volume of 4 μL of peptide mixtures was injected onto a 5 mm long × 300 μm C18 PepMap 100 μ-precolumn, 5 μm, 100 Å (Thermo-Scientific) and then to a 150 mm × 75 μm Acclaim PepMap 100 NanoViper C18, 3 μm, 100 Å (Thermo-scientific) column. The MS scan range was *m/z* 350 to 1,600, and the spray voltage was 1,500 V. Top 10 precursor ions were selected in the MS scan by Orbitrap with a resolution *r* = 60,000 for fragmentation in the linear ion trap using collision-induced dissociation. Normalized collision-induced dissociation was set at 35.0. Spectra were submitted to a local MASCOT (Matrix Science, London, United Kingdom) server and searched against arabidopsis in the TAIR (release 10), with a precursor mass tolerance of 10 ppm, a fragment ion mass tolerance of 0.5 Da, and strict trypsin specificity allowing up to one missed cleavage, carbamidomethyl modification on cysteine residues as fixed modification and oxidation of methionine residues, and citrullination (also known as deimination) of arginine residues as variable modifications. Notably, an additional variable modification, deimination of Asn/Gln, was assessed to rule out mis-assignment of the citrullination modification. In addition, the neutral loss of 43 Da was manually assessed from the MS peak fragmentation to validate the assigned citrullination modification. Proteins were considered positively identified if the Mascot score was over the 95% confidence limit corresponding to a score ≤ 32, and peptide was considered citrullinated if the Mascot ion score was ≤30.

### Data Analysis

WebLogo3^2^ was used to predict the specificity of citrullination on the identified citrullination candidates. A gene ontology (GO) analysis toolkit and database for agricultural community, AgriGO^3^, was used for GO-based analysis to detect enriched cellular components, biological processes, and molecular functions from the set of citrullinated candidate proteins.

### Structural Analysis

Amino acid consensus motifs of catalytic centers were obtained as first employed in the search for plant nucleotide cyclases (Ludidi and Gehring, 2003; Gehring, 2010), and the motif searches of Swiss-Prot were done online^4^. The crystal structure of *A. thaliana* agmatine deiminase At5g08170 was obtained from a protein data bank (PDB ID: 3H7K), and the location of the arginine citrullination catalytic center was identified and visualized using UCSF Chimera (ver. 1.10.1; Pettersen et al., 2004). Two selected citrullinated proteins At2g21450 and At4g00830 were modeled against the chain K of an ATPase domain of a chromatin remodeling factor (PDB ID: 6PWF) and the chain D of decaheme c-type cytochrome (PDB ID: 6R2K), respectively, using the Modeler (ver. 9.14) software (Sali and Blundell, 1993). The citrullinated arginines in the generated models were visualized and assessed for their ability to spatially fit the catalytic center of At5g08170. Docking simulations of the citrullinated arginines from the two peptides to the catalytic center of At5g08170 were performed using AutoDock Vina (ver. 1.1.2; Sali and Blundell, 1993; Trott and Olson, 2010). All bonds in the arginines were set as nonrotatable to keep their poses similar to that in the peptides. The arginine docking poses were analyzed, and all images were created by UCSF Chimera (ver. 1.10.1; Pettersen et al., 2004). Chimera was developed by the Resource for Biocomputing, Visualization, and Informatics at the University of California, San Francisco (supported by NIGMS P41-GM103311).

### Cloning and Expression of At5g08170

Total RNA from arabidopsis (Col-0) leaves was extracted using an RNeasy Plant Mini Kit (Qiagen) followed by the synthesis of cDNA using the SuperScript^TM^ II Reverse Transcriptase system (Invitrogen). Full-length At5g08170 was then detected and amplified by polymerase chain reaction (PCR) using the forward primer (5′- ATGGAGGAGTCACGAGAATCG-3′) and reverse primer (5′-TCAGTGGCCATTTTCGGC-3′). The PCR-amplified gene was cloned into pCR8^TM^/GW/TOPO^§^ plasmid prior to cloning it into a pDEST^TM^ 17 destination vector using GATWAY cloning technology (Invitrogen). Plasmid containing the full-length gene was then transformed into *E. coli* BL-21 A1 cells (Invitrogen). The expression of recombinant protein was induced by 0.2% (w/v) L-Arabinose (Sigma Aldrich). The expressed protein was purified as His-tagged fusion protein using Ni-NTA agarose beads (Qiagen) and analyzed by SDS-PAGE.

### Enzyme Activity Analysis via Mass Spectrometry

Two synthetic peptides from two of the identified citrullinated proteins, LIF2 and chromatin remodeling 34, were designed and purchased (Thermo Fisher Scientific). The selected peptides were designed to ensure that all peptides had at least one arginine or lysine to confirm the success of digestion. A tryptic digestion was performed following a citrullination assay that was performed in 50 mM CHES, pH 9.5, 5 mM CaCl_2_, 10 mM DTT, 50 μg of different substrates (including a CHES buffer as the control, the two synthetic peptides and fibrinogen), and 50 μg of purified At5g01870 in a total volume of 200 μL in a microcentrifuge tube. Notably, auto-citrullination of the agmatine deiminase in the presence and absence of calcium was also assessed by tandem mass spectrometry as described earlier.

## Data Availability Statement

The original contributions presented in the study are included in the article/Supplementary Material; further inquiries can be directed to the corresponding author/s.

## Author Contributions

GE conceived the project. CM and CG planned the experiments. CM and LT did the experiments. AW did the structural modeling. All authors contributed to the writing of the manuscript.

## Conflict of Interest

The authors declare that the research was conducted in the absence of any commercial or financial relationships that could be construed as a potential conflict of interest.

## Footnotes

1 see http://bar.utoronto.ca

2 http://weblogo.threeplusone.com, 2015.

3 http://bioinfo.cau.edu.cn/agriGO/

4 https://www.genome.jp/tools/motif/MOTIF2.html
