# Supplementary material for: Citrullination of Proteins as a Specific Response Mechanism in Plants
Source: Front Plant Sci. 2021 Apr 8;12:638392. doi: 10.3389/fpls.2021.638392 (PMC8060559; doi:10.3389/fpls.2021.638392)
Supplement: Supplementary file 1 [file Data_Sheet_1.pdf]

Supplementary Material

1 Supplementary Figures and Tables

1.1 Supplementary Figure 1

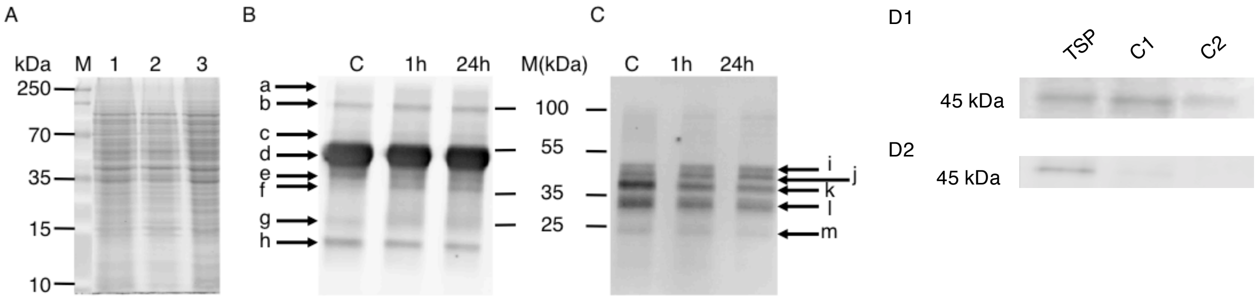

Supplementary Figure 1. One-dimensional gel electrophoresis scans showing nuclear enriched proteins following Coomassie staining (**A**), western blots of immunoprecipitated citrullinated candidate proteins (**B** and **C**). In **A**, the first lane represents the protein ladder (M), lane 1 is the nuclear extract from the control samples of Arabidopsis cell suspension culture, lane 2 is nuclear extract from 1-hour cold treated Arabidopsis cell suspension culture sample and lane 3 is the nuclear extract from 24 hour cold treated Arabidopsis cell suspension culture sample. For the Western blots the three lanes represent control (C), and one hour post cold treatment (1h) and 24 hours post cold treatment (24h). **B** shows a western blot where the nuclear extract was incubated overnight with the anti-citrulline antibody and then conjugated with anti-citrulline IgG – Protein A beads. **C** shows a western blot where the anti-citrulline antibody was conjugated with anti-citrulline IgG – Protein A beads and then incubated with the nuclear extract for 10 minutes according to the manufacturer's instruction (see main text, Materials and Methods, Section 3.3). In **B**, **a**=AAA-type ATPase family protein, target of rapamycin, methyltransferase A70, far-red impaired responsive, calcium exchanger 7; **b**=chromatin remodeling 34, unknown protein, ARM repeat superfamily protein; **c**= no positive identification (ND); **d**=RNA-binding (RRM/RBD/RNP motifs) family protein; **e**=Unknown protein; **f**=AAA-type ATPase family protein; **g**=chromatin remodeling 34 (fragment), GDA1/CD39 nucleoside phosphatase; **h**=RING/FYVE/PHD zinc finger superfamily protein. In **C**; **i**=F-box family protein; **j**=decapping 5, thioredoxin family protein; **k**=AAA-type ATPase family protein, RNA-binding (RRM/RBD/RNP motifs) family protein; **l**=peroxidase superfamily protein; **m**= chromatin remodeling 34 (fragment). It is worth noting is that the molecular mass of chromatin remodeling 34 is about 94kDa and in "B", band (b) reflects the approximate actual molecular mass of the entire protein while the IDs from bands (g in "B") and (m in "C") are truncated versions of the protein and likely the helicase ATP-binding domain. β-actin was used as a control for the IPs (**D**). **D1** represents the anti-β-actin western blot on the total soluble extracts (TSP), TSP of control rep1 (C1) and control rep 2 (C2). **D2** shows anti-β-actin western blot on the TSP, and C1 and C2 after anti-citrulline IgGIPs.

1.2 Supplementary Figure 2

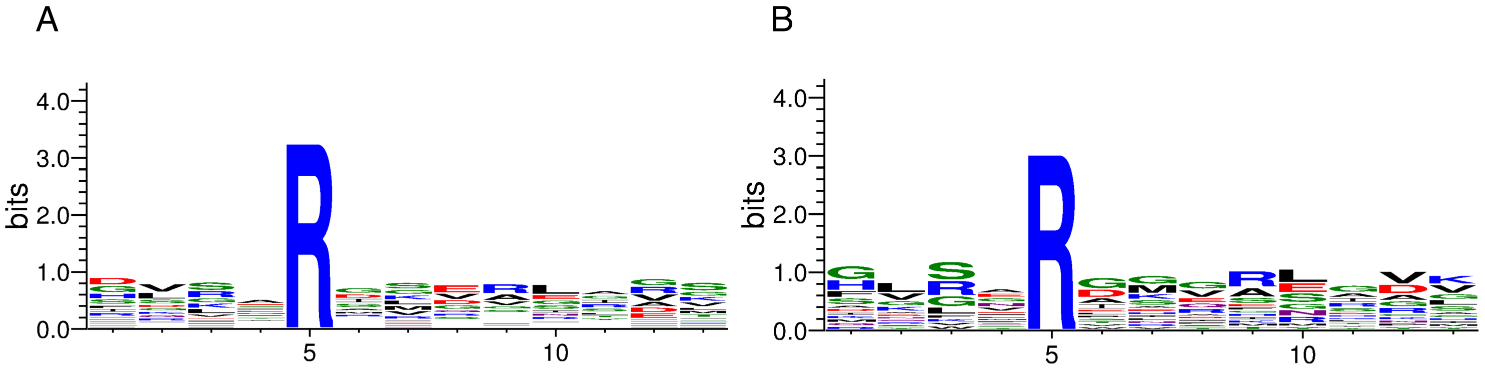

Supplementary Figure 2. (**A**) The sequence logo representation of the citrullinated arginines in all peptides and (**B**) representation of the citrullinated arginines that are not located on the N-terminus or C-terminus of the identified peptide. The citrullinated residue (R) in the logo is flanked by four amino acids on the N- terminus and eight amino acids on the C-terminus. The logo was generated using the WebLogo 3.4 (http://weblogo.threeplusone.com/create.cgi).

1.3 Supplementary Figure 3

```
WP_003291896.1             ------------------------------------------------------------ 0
WP_173063483.1             ------------------------------------------------------------ 0
XP_012894396.1             ------------------------------------------------------------ 0
XP_021731209.1             ------------------------------------------------------------ 0
XP_010423126.1             ------------------------------------------------------------ 0
NP_196434.1                ------------------------------------------------------------ 0
sp|Q9Y2J8|PADI2_HUMAN      MLRERTVRLQYGSRVEAVYVLGTYLWTDVYSAAPAGAQTFSLKHSEHVWVEVVRDGEAEE 60
sp|Q9UM07|PADI4_HUMAN      MAQGTLIRVTPEQPTHAVCVLGTLTQLDICSSAPEDCTSFSINASPGVVVDIAH-GPPAK 59

WP_003291896.1             ------------------------------------------------------------ 0
WP_173063483.1             ------------------------------------------------------------ 0
XP_012894396.1             ------------------------------------------------------------ 0
XP_021731209.1             ------------------------------------------------------------ 0
XP_010423126.1             ------------------------------------------------------------ 0
NP_196434.1                ------------------------------------------------------------ 0
sp|Q9Y2J8|PADI2_HUMAN      VATNGKQRWLLSPSTTLRVTMSQASTEASSDKVTVNYYDEEGSIPIDQAGLFLTAIEISL 120
sp|Q9UM07|PADI4_HUMAN      KKSTGSSTWPLDPGVEVTLTMKVASGSTGDQKVQISYYGPKT--PPVKALLYLTGVEISL 117

WP_003291896.1             ------------------------------------------------------------ 0
WP_173063483.1             ------------------------------------------------------------ 0
XP_012894396.1             ------------------------------------------------------------ 0
XP_021731209.1             ------------------------------------------------------------ 0
XP_010423126.1             ------------------------------------------------------------ 0
NP_196434.1                ------------------------------------------------------------ 0
sp|Q9Y2J8|PADI2_HUMAN      DVDADRDGVVEKNN--PKKASWTWGPEGQGAILLVNCDRETPWLPKEDCRDEKVYSKEDL 178
sp|Q9UM07|PADI4_HUMAN      CADITRTGKVKPTRAVKDQRTWTWGPCGQGAILLVNCDRDNLESSAMDCEDDEVLDSEDL 177

WP_003291896.1             -----------------------------------------------------------M 1
WP_173063483.1             -----------------------------------------------------------M 1
XP_012894396.1             -----------------------------------------------------------M 1
XP_021731209.1             ------------------------------------------------------------ 0
XP_010423126.1             ------------------------------------------------------------ 0
NP_196434.1                -----------------------------------------------------------M 1
sp|Q9Y2J8|PADI2_HUMAN      KDMSQMILRTKGPDRLPAGYEIVLYISMSDSDKVGVFYVENPFFGQRYIHILGRRKLYHV 238
sp|Q9UM07|PADI4_HUMAN      QDMSLMTLSTKTPKDFFTNHTLVLHVARSEMDKVRVFQATRGKLSSKCSVVLGPKWPSHY 237

WP_003291896.1             TTLSSTPRQDGYYMPAEW--------------------------APHSQTWMVWPQRPDN 35
WP_173063483.1             KTLLSTPKKDGYRMPGEF--------------------------EYHDGCWMIWPERTDN 35
XP_012894396.1             SVLPGFAADDGFKAPAEW--------------------------DVHEQTWMGFPQRPDI 35
XP_021731209.1             MEEIGTPAENGYCMPAEW--------------------------EPHSHCWLGWPERPDN 34
XP_010423126.1             -MVEESPAEHGFYMPAEW--------------------------EPHAQTWIGWPERQDN 33
NP_196434.1                EESRESPAEHGYYMPAEW--------------------------DSHAQTWIGWPERQDN 35
sp|Q9Y2J8|PADI2_HUMAN      VKYTGGSAELLFFVEGLCFPDEGFSGLVSIHVSLLEYMAQDIPLTPIFTDTVIFRIAPWI 298
sp|Q9UM07|PADI4_HUMAN      LMVPGGKHNMDFYVEALAFPDTDFPGLITLTISLLDTSNLELPEAVVFQDSVVFRVAPWI 297
                                   .  :   .                                   : :

WP_003291896.1             WRDNATPAQAAFTAVAKAIARFEPVTVCASAEQYLAARAALDDPRIRVVEMSTDDAWVRD 95
WP_173063483.1             WRNGAKPAQHAFVDVAKAISEFEPVTMCVNQHQYVNARHMLPD-YVRVVEMATNDAWMRD 94
XP_012894396.1             WRENAAPAQKVFANVANAIARFEPVTVCAPKELYTVARSLLDK-NVRVVEMSMNDSWFRD 94
XP_021731209.1             WRDNAVAAQNVFAKVATAISKFEPVTVCASPAQWTNARSQLPP-NVRVVEMSMNDSWFRD 93
XP_010423126.1             WRHDALPAQRVFVDVAKAISKFEPVTVCASPAQWENAMKQLPE-DIRVVEMSMNDSWFRD 92
NP_196434.1                WRHNALPAQRVFADVAKAISKFEPVTVCASPAQWENARKQLPE-DIRVVEMSMNDSWFRD 94
sp|Q9Y2J8|PADI2_HUMAN      MTPNILPPVSVFVCCMKDNYLFLKE-------VKNLVEKTNCELKVCFQYLNRGDRWIQD 351
sp|Q9UM07|PADI4_HUMAN      MTPNTQPPQEVYACSIFENEDFLKS-------VTTLAMKAKCKLTICPEEENMDDQWMQD 350
                              .      .:.        *              .        :      .* *.:*

WP_003291896.1             TGPTFVIDD---------------------HGGLRGVDWTFNAWGGDDGGLYSDWQRDDE 134
WP_173063483.1             VGPTFVVHDG--------------------TGDIRGVDWAFNAWGGLIDGLYFPWDEDDR 134
XP 012894396.1             TGAIFVKNEE---------------------GVVRGTNWLFNSWGGLNGGCYDYWEDDLL 133
```

```
_
XP_021731209.1             SGPTFVVRKH----------ISSS---GATLKSLAGIDWNFNSWGGVDDGCYTDWSHDIL 140
XP_010423126.1             SGPTFIVRKR----------PLKL---SSLNRNIAGIDWNFNAWGGACDGCYNDWSHDLL 139
NP_196434.1                SGPTFIVRKR----------PVKL---SSLNRNIAGIDWNFNAWGGANDGCYNDWSHDLL 141
sp|Q9Y2J8|PADI2_HUMAN      EIEFGYIEAPHKGFPVVLDSPRDGNLKDFPVKELLGPDFGYVTREPLFESVTSLDS---- 407
sp|Q9UM07|PADI4_HUMAN      EMEIGYIQAPHKTLPVVFDSPRNRGLKEFPIKRVMGPDFGYVTRGPQTGGISGLDS---- 406
                                                            : * :: : :      .     .

WP_003291896.1             VARKILEVEHCDRYRTEGFVLEGGSIHVDGEGTLITTEECLLNRNRNPHLSREEIETVLR 194
WP_173063483.1             VAEKICDLEGKDRYRLNNFVLEGGSIHVDGEGTVITTEECLLSSGRNPSLSKQEIEETLK 194
XP_012894396.1             VAGKMCNIERVPYYKY-NMILEGGSISFDGEGTLLTTEECLLNPNRNPSMTKEQIEAELK 192
XP_021731209.1             VSRKILEIEKLPRFPH-TMILEGGSIHVDGEGTCLTTEECLLNKNRNPHMSKEQIEDNLK 199
XP_010423126.1             VSKKILAVERIPRFQH-SMILEGGSIHVDGEGTCLVTEECLLHENRNPHMCKEQIEEELK 198
NP_196434.1                VSRKILALERIPRFQH-SMILEGGSIHVDGEGTCLVTEECLLNKNRNPHMSKEQIEEELK 200
sp|Q9Y2J8|PADI2_HUMAN      ----FGNLEVSPP--------------VTVNGKTYPLGRILIGSS-FPLSGGRRMTKVVR 448
sp|Q9UM07|PADI4_HUMAN      ----FGNLEVSPP--------------VTVRGKEYPLGRILFGDSCYPSNDSRQMHQALQ 448
                               :  :*                  .  .*.     . *:  .  *    ..:   ::

WP_003291896.1             DHLAVDTIIWLPHGLFND-ETDGHVDNFCCFVRPGE----VLLAWTDDANDPNFERCQAA 249
WP_173063483.1             EYLGAEKVIWLKRGIYLD-ETNGHVDNICNFVRPGE----VVLAWTDDESDPQYEISKEC 249
XP_012894396.1             RGLGVEKVIWLPNGLFGDVDTNGHVDNFCVFARPGE----VLLSWTDDEKDPQYPISQHA 248
XP_021731209.1             EYLGVQKIIWLPRGLHGDDDTNGHIDNMCCFVKPGV----VLLSWTDDESDPHYERALEA 255
XP_010423126.1             KYLGVESFIWIPRGLYGDEDTNGHIDNMCCFAKPGV----VLLSWTDDETDPQYERSVEA 254
NP_196434.1                KYLGVQSFIWLPRGLYGDEDTNGHIDNMCCFARPGV----VLLSWTDDETDPQYERSVEA 256
sp|Q9Y2J8|PADI2_HUMAN      DFLKAQQV-QAPVELYSDWLTVGHVDEFMSFVPIPGTKKFLLLM----------ASTSAC 497
sp|Q9UM07|PADI4_HUMAN      DFLSAQQV-QAPVKLYSDWLSVGHVDEFLSFVPAPDRKGFRLLL----------ASPRSC 497
                             *   .: .      :. *  : **:*::  *.         :*                .

WP_003291896.1             MAVLQTA-RDARGRALTVHRMPI-------------PG---------------------- 273
WP_173063483.1             YEILTNE-FDAKGRKLTVHKLYL-------------PS---------------------- 273
XP_012894396.1             YKLLEAA-TDAKGRHLKIHKLHI-------------PS---------------------- 272
XP_021731209.1             LSVLSST-TDANGRKLEVVKLHI-------------PC---------------------- 279
XP_010423126.1             LSVFSKS-FDARGRKIEVIKLHI-------------PG---------------------- 278
NP_196434.1                LSVLSNS-IDARGRKIQVIKLYI-------------PE---------------------- 280
sp|Q9Y2J8|PADI2_HUMAN      YKLFREKQKDGHGEAIMFKGLGGMSSKRITINKILSNESLVQENLYFQRCLDWNRDILKK 557
sp|Q9UM07|PADI4_HUMAN      YKLFQEQQNEGHGEALLFEGIKKKK--QQKIKNILSNKTLREHNSFVERCIDWNRELLKR 555
                             ::     :..*. : .  :

WP_003291896.1             PLHATEQECAGVLPLDGSQPRDPSIRLAGSYVNFLIVNGGIIAPA-FGDPL-D----AEA 327
WP_173063483.1             PILITKEESEGVDTVDGTLPRVEGDRLAASYANYYTANGGVVIPQ-FNDPS-D----EKA 327
XP_012894396.1             DIIRTPEEFAGLTQEEGTIEREENQRLPASYVNFYFANGAIISPC-FGVKE-D----EMA 326
XP_021731209.1             PLYMTDEESAGIIQDGEAKPREPGTRLAASYVNFYIANGGIIAPQ-FGDKKWD----NEA 334
XP_010423126.1             PLYMTEEEASGITQEGEAIPRIAGTRLAASYVNFYIANGGIIVPK-FGDPKRD----EEA 333
NP_196434.1                PLYMTEEESSGITQDGEAIPRLAGTRLAASYVNFYIANGGIIAPQ-FGDPIRD----KEA 335
sp|Q9Y2J8|PADI2_HUMAN      ELGLTEQDIIDLPALFKMDEDHRARAFFPNMVNMIVLDKDLGIPKPFGPQVEEECCLEMH 617
sp|Q9UM07|PADI4_HUMAN      ELGLAESDIIDIPQLFKLKEFSKAEAFFPNMVNMLVLGKHLGIPKPFGPVINGRCCLEEK 615
                            :  : .:  .:              :  . .*    .  :  *  *.

WP_003291896.1             ERILVQLFPEHEVVMV-AGREILLGGGNIHCITQQQPAPRPR-----------        368
WP_173063483.1             LALFSELYPERKVVGV-YAREILLGGGNIHCITQQQPLAIPAIKPLELVGAR-        378
XP_012894396.1             RKVFQEVFPEREVVMV-PTREVILGGGNIHCITQQQPKGVKA-----------        367
XP_021731209.1             VRVLSEAFPDYEVVKIEGAREIVLAGGNIHCITQQQPSMK-------------        374
XP_010423126.1             IRVLSETFPHHSVVGIENAREIVLAGGNIHCITQQQPVEPSSVAENGHAPLRD        386
NP_196434.1                IRVLSDTFPHHSVVGIENAREIVLAGGNIHCITQQQPAEPTSVAENGH-----        383
sp|Q9Y2J8|PADI2_HUMAN      VRGLLEPLG-LECTFIDDISAYHKFLGEVHCGTNVRRKPFTF-KWWHMVP---        665
sp|Q9UM07|PADI4_HUMAN      VCSLLEPLG-LQCTFINDFFTYHIRHGEVHCGTNVRRKPFSF-KWWNMVP---        663
                              : :     . . :          *::** *: :
```

Supplementary Figure 3. Alignment of arginine deiminases: WP_003291896.1 [*Pseudomonas stutzeri*], WP_173063483.1 [Bacillus sp. BRMEA1], XP_012894396.1 *Blastocystis hominis*], XP_021731209.1 [*Chenopodium quinoa*], XP_010423126.1 [*Camelina sativa*], NP_196434.1 [*Arabidopsis thaliana*], sp|Q9Y2J8|PADI2_HUMAN Protein-arginine deiminase type-2, sp|Q9UM07|PADI4_HUMAN Protein-arginine deiminase type-4.

1.4 Supplementary Figure 4

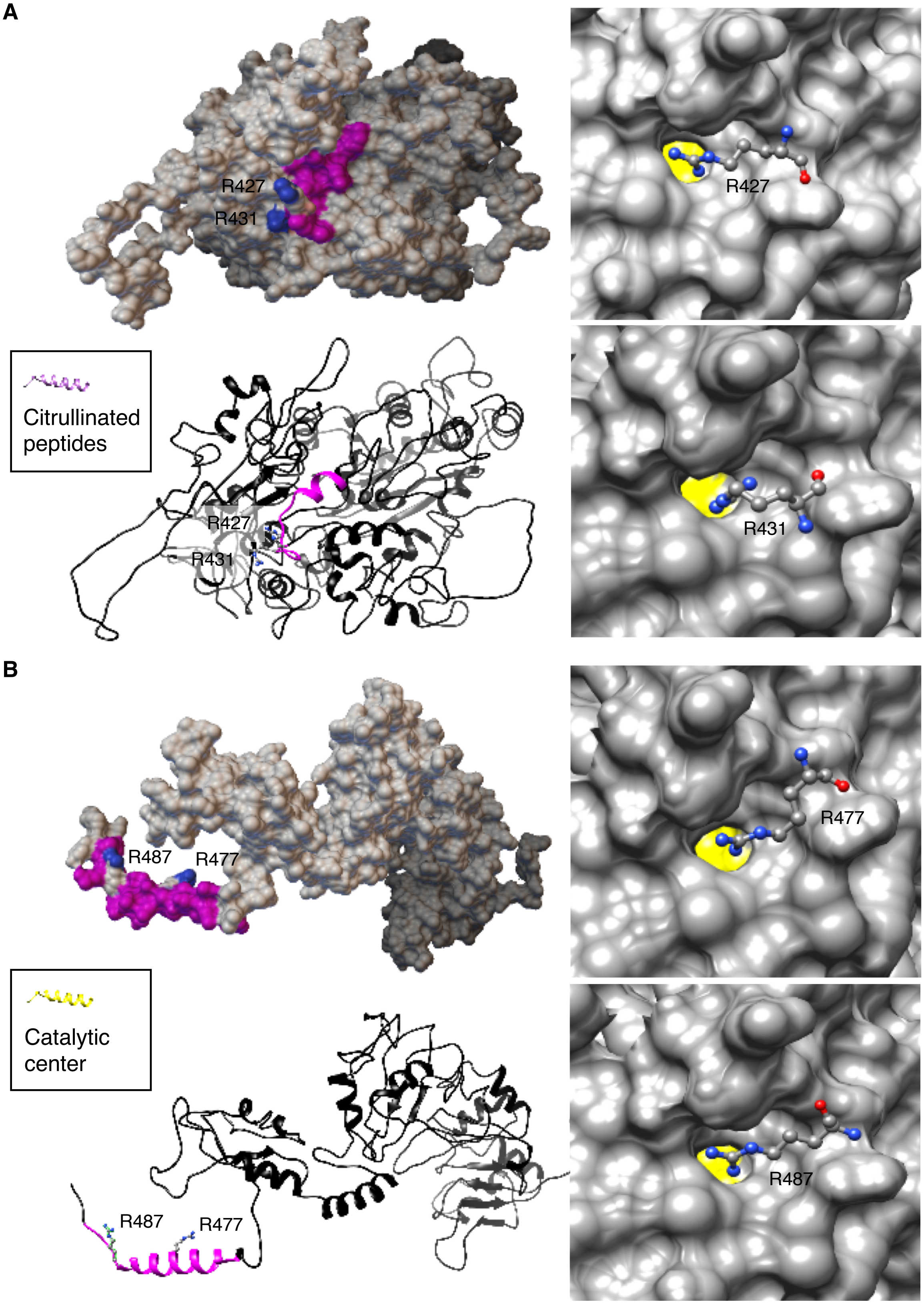

Supplementary Figure 4. Computational assessment of the citrullinated arginines of two selected proteins (**A**) At2g21450 and (**B**) At4g00830. At2g21450 and At4g00830 were modeled against the chain K of a ATPase domain of a chromatin remodeling factor (PDB ID: 6PWF) and the chain D of decaheme c-type cytochrome (PDB ID: 6R2K) respectively using the Modeller (ver. 9.14) software. The citrullinated arginines (colored according to surface charges) in the generated models were visualized and assessed for their ability to spatially fit the catalytic center of At5g08170. Citrullinated peptides were colored magenta and citrullinated arginine residues are all solvent exposed as shown in the ribbon and surface models of At2g21450 and At4g00830 respectively (left panels). Individual citrullinated residue: R427 and R431 of At2g21450, and R477 and R487 of At4g00830, were respectively docked at the catalytic center cavity of At5g08170, keeping all bonds in the R ligand non-rotatable so that their poses in the generated 3D models are retained. All citrullinated arginines docked at the catalytic cavity in a binding pose deemed suitable for catalysis i.e., with the amine rich region pointing into the cavity, as shown in the surface models (right panels). All docking simulations were performed using AutoDock Vina (ver. 1.1.2). Docking poses were analyzed, and all images created using UCSF Chimera (ver. 1.10.1). Chimera was developed by the Resource for Biocomputing, Visualization, and Informatics at the University of California, San Francisco (supported by NIGMS P41-GM103311).

1.5 Supplementary Figure 5

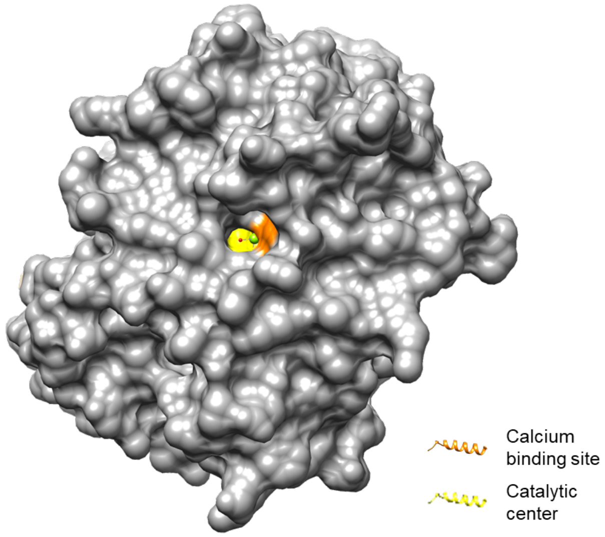

Supplementary Figure 5. Predicted calcium binding site of At5g08170. There were a total of 14 possible Ca^{2+} binding sites in At5g08170 predicted by the MIB: Metal Ion-Binding site prediction and docking server available at: http://bioinfo.cmu.edu.tw/MIB [Lin YF, Cheng CW, Shih CS, Hwang JK, Yu CS, Lu CH. MIB: Metal Ion-Binding Site Prediction and Docking Server. J Chem Inf Model. 2016 Dec 27;56(12):2287-2291. doi: 10.1021/acs.jcim.6b00407], with P97, G117 and D119 binding residues forming the binding site (orange) that is closest to the catalytic center (R93 – G96) (yellow). Structural analysis was performed using UCSF Chimera (ver. 1.10.1). Chimera was developed by the Resource for Biocomputing, Visualization, and Informatics at the University of California, San Francisco (supported by NIGMS P41-GM103311).

1.6 Supplementary Table 1

A. Commonly citrullinated peptides following citrullination of fibrinogen with plant agmatine deiminase

Protein accession	Protein description	Peptide sequence	Literature*
gi|237823914	chain A, Human fibrinogen	ADSGEGDFLAEGGGVrGPR	X
gi|182439	fibrinogen gamma chain	ANQQFLVYCEIDGSGNGWTVFQKr	X
gi|237823915	chain B, Human fibrinogen	EEAPSLrPAPPPISGGGYR	X
gi|4503689	fibrinogen α-E preproprotein	GDFSSANNrDNTYNR	X
gi|237823915	chain B, Human fibrinogen	GGETSEMYLIQPDSSVKPYrVYCDMNTENGGWTVIQNr	X(C-Term.)
gi|4503689	fibrinogen α-E preproprotein	GGSTSYGTGSETESPrNPSSAGSWNSGSSGPGSTGNr	X
gi|4503689	fibrinogen α-E preproprotein	GGSTSYGTGSETESPrNPSSAGSWNSGSSGPGSTGNR	X
gi|4503689	fibrinogen α-E preproprotein	HrHPDEAAFFDTASTGK	X
gi|237823916	chain C, Human fibrinogen	IHLISTQSAIPYALrVELEDWNGR	
gi|182439	fibrinogen gamma chain	IHLISTQSAIPYALrVELEDWNGR	
gi|182439	fibrinogen gamma chain	IIPFNrLTIGEGQQHHLGGAK	X
gi|4503689	fibrinogen α-E preproprotein	QFTSSTSYNrGDSTFESK	X
gi|4503689	fibrinogen α-E preproprotein	TFPGFFSPMLGEFVSETESrGSESGIFTNTK	X
gi|182439	fibrinogen gamma chain	VELEDWNGrTSTADYAMFK	X
gi|237823915	chain B, Human fibrinogen	VYCDMNTENGGWTVIQNrQDGSVDFGR	X
gi|237823915	chain B, Human fibrinogen	VYCDMNTENGGWTVIQNrQDGSVDFGr	

B. LHP1-INTERACTING FACTOR 2 RNA binding protein peptides that are citrullinated

Protein accession	Protein description	Peptide sequence	Peptide score
AT4G00830.1	RNA-binding protein	NDrNNGSSGGSGRDNSHEHDGNR	50
AT4G00830.1	RNA-binding protein	NDrNNGSSGGSGrDNSHEHDGNR	50

*- **Fibrinogen peptides citrullinated by rmPAD2 in:** van Beers, J.J., Raijmakers, R., Alexander, LE. *et al.* Mapping of citrullinated fibrinogen B-cell epitopes in rheumatoid arthritis by imaging surface plasmon resonance. *Arthritis Res Ther* 12, R219 (2010). https://doi.org/10.1186/ar3205

- The red colour denotes citrullinated residues.

1.7 Supplementary Table 2

Auto-citrullination of agmatine deiminase in the presence or absence of calcium

Agmatine peptide	Citrullinated site(s)	Stoichiometry (%; A,B)
ESPAEHGYYMPAEWDSHAQTWIGWPErQDNWr	32, 37	13, 25
ESPAEHGYYMPAEWDSHAQTWIGWPErQDNWR	32	30, 37
FEPVTVCASPAQWENArK	73	11, 17
GLYGDEDTNGHIDNMCCFArPGVVLLSWTDDETDPQYEr	233, 252	14, 18
GLYGDEDTNGHIDNMCCFArPGVVLLSWTDDETDPQYER	233	49, 35
LAASYVNFYIANGGIIAPQFGDPIrDK	331	20, 43
LYIPEPLYMTEEESSGITQDGEAIPrLAGTr	301, 306	11, 33
LYIPEPLYMTEEESSGITQDGEAIPrLAGTR	301	33
NIAGIDWNFNAWGGANDGCYNDWSHDLLVSrK	144	10, 18
QDNWrHNALPAQR	37	14, 19
QLPEDIrVVEMSMNDSWFR	81	11, 2
QLPEDIrVVEmSMNDSWFR	81	2
VVEMSMNDSWFrDSGPTFIVR	93	44, 50

A: Agmatine – $CaCl_2$; B: Agmatine + $CaCl_2$
